# Butyrate inhibits IL-1β-induced inflammatory gene expression by suppression of NF-κB activity in pancreatic beta cells

**DOI:** 10.1016/j.jbc.2022.102312

**Published:** 2022-07-31

**Authors:** Signe Schultz Pedersen, Michala Prause, Kristine Williams, Romain Barrès, Nils Billestrup

**Affiliations:** 1 Department of Biomedical Sciences, Faculty of Health and Medical Sciences, University of Copenhagen, Copenhagen, Denmark; 2 Novo Nordisk Foundation Center for Basic Metabolic Research, Faculty of Health and Medical Sciences, University of Copenhagen, Copenhagen, Denmark; 3 Institut de Pharmacologie Moléculaire et Cellulaire, Université Côte d'Azur and CNRS, Valbonne, France

**Keywords:** butyrate, beta cells, inflammation, interleukin-1β, NF-κB transcription factor, acetylation, ChIP, chromatin immunoprecipitation, CXCL1, (C-X-C motif) ligand 1, EMSA, electrophoretic mobility shift assay, FFAR, free fatty receptor, HDAC, histone deacetylase, IL-1β, interleukin-1β, iNOS, inducible nitric oxide synthase, NO, nitric oxide, NF-κB, nuclear factor-κB, P/S, penicillin/streptomycin, RNAPII, RNA polymerase II, SAHA, Suberoylanilide Hydroxamic Acid, T2D, type 2 diabetes

## Abstract

Cytokine-induced beta cell dysfunction is a hallmark of type 2 diabetes (T2D). Chronic exposure of beta cells to inflammatory cytokines affects gene expression and impairs insulin secretion. Thus, identification of anti-inflammatory factors that preserve beta cell function represents an opportunity to prevent or treat T2D. Butyrate is a gut microbial metabolite with anti-inflammatory properties for which we recently showed a role in preventing interleukin-1β (IL-1β)-induced beta cell dysfunction, but how prevention is accomplished is unclear. Here, we investigated the mechanisms by which butyrate exerts anti-inflammatory activity in beta cells. We exposed mouse islets and INS-1E cells to a low dose of IL-1β and/or butyrate and measured expression of inflammatory genes and nitric oxide (NO) production. Additionally, we explored the molecular mechanisms underlying butyrate activity by dissecting the activation of the nuclear factor-κB (NF-κB) pathway. We found that butyrate suppressed IL-1β-induced expression of inflammatory genes, such as *Nos2*, *Cxcl1*, and *Ptgs2*, and reduced NO production. Butyrate did not inhibit IκBα degradation nor NF-κB p65 nuclear translocation. Furthermore, butyrate did not affect binding of NF-κB p65 to target sequences in synthetic DNA but inhibited NF-κB p65 binding and RNA polymerase II recruitment to inflammatory gene promoters in the context of native DNA. We found this was concurrent with increased acetylation of NF-κB p65 and histone H4, suggesting butyrate affects NF-κB activity *via* inhibition of histone deacetylases. Together, our results show butyrate inhibits IL-1β-induced inflammatory gene expression and NO production through suppression of NF-κB activation and thereby possibly preserves beta cell function.

Type 2 diabetes (T2D) is a growing global health problem. It is characterized by chronic hyperglycemia with common complications such as cardiovascular diseases, kidney, and neuronal damage (1). T2D develops when insulin-producing pancreatic beta cells fail to compensate for insulin resistance and is often associated with obesity and chronic systemic low-grade inflammation (2). Increasing evidence suggests that beta cell failure is due to dysfunction and loss of beta cell identity, a process described as *dedifferentiation*, rather than loss of beta cell mass (3). Thus, preventing or reversing beta cell dysfunction is of great interest to maintain or improve glucose homeostasis.

Among several factors, proinflammatory cytokines impair beta cell function in mice and humans (4, 5). T2D individuals have immune cell infiltration in pancreatic islets (6, 7, 8, 9) and higher levels of circulating proinflammatory cytokines compared to healthy individuals (10). Both islets and infiltrating immune cells in the pancreas produce cytokines and chemokines that exacerbate local inflammation (4, 11). In particular, the proinflammatory cytokine interleukin-1β (IL-1β) reduces beta cell insulin secretion, viability, and induces dedifferentiation (6, 12, 13). Blockade of the IL-1β receptor improves hyperglycemia, beta cell function, and reduces markers associated with inflammation in T2D individuals, suggesting that IL-1β plays a role in T2D (14).

Beta cells exposed to IL-1β undergo extensive gene expression changes, which include increased expression of genes involved in inflammatory processes and reduced expression of genes controlling beta cell function (12, 15). Among other genes, IL-1β induces the expression of the gene *Nos2* encoding the enzyme-inducible nitric oxide synthase (iNOS) (16, 17) and various chemokines such as (C-X-C motif) ligand 1 (*Cxcl1*) that recruit and activate certain immune cells (18). iNOS produces nitric oxide (NO) that in high concentrations inhibits glucose-stimulated insulin secretion in beta cell lines (13), rodent (19, 20, 21) and human islets (22, 23) *e.g.*, *via* inactivation of mitochondrial aconitase resulting in decreased oxidative metabolism and depletion of ATP (17). Moreover, NO induces DNA-damage and regulates the expression and activity of several proteins, thus being a major mediator of IL-1β-induced beta cell failure (13, 15, 24, 25). However, NO-independent mechanisms also exist, and especially human islets seem to be less sensitive to NO (26).

The activation of transcription factors, mainly nuclear factor-κB (NF-κB), mediates IL-1β-induced gene expression (13, 25, 27). NF-κB is a dimeric transcription factor consisting of homodimers or heterodimers of p50 and/or p65. In its inactive state, NF-κB is bound to IκB inhibitory proteins in the cytoplasm. Upon stimuli by cytokines such as IL-1β, the IκB kinase phosphorylates IκB, which in turn induces ubiquitin-mediated proteasomal degradation of IκB. This allows nuclear translocation of NF-κB that binds specific DNA elements and subsequent assembly of the transcriptional machinery which initiates transcription (28). Regulation of NF-κB activity occurs at multiple levels and thus, identifying inhibitors of the activation has the potential to be an effective approach to prevent IL-1β-induced beta cell dysfunction.

Short-chain fatty acids, predominately butyrate, propionate, and acetate, are products of bacterial fermentation of dietary fiber in the gut (29). Changes in the gut microbiota and their metabolites have been linked to T2D. Indeed, individuals with T2D have a lower abundance of butyrate-producing bacteria in the gut compared to healthy controls (30, 31, 32, 33, 34). As diet supplementation with butyrate in animal models of diabetes has shown promising results on circulating glucose levels, these results suggest that butyrate may play a role in glucose homeostasis (35, 36, 37, 38). In various cell types, butyrate inhibits inflammation, NF-κB activation, and iNOS *in vitro* (39, 40, 41, 42, 43, 44, 45). Two main mechanisms have been suggested: (1) inhibition of histone deacetylases (HDACs) leading to increased acetylation of histones and nonhistone proteins and (2) binding to the free fatty receptor 2 (FFAR2) and FFAR3 (46). Beta cells express both FFAR2 and FFAR3 (47) and HDAC inhibitors have proven to be efficient in preventing cytokine-induced beta cell dysfunction both *in vitro* (48, 49) and *in vivo* (50, 51). We and others have shown that butyrate also directly affects beta cell function both in presence and absence of inflammatory stress (47, 52, 53, 54). For example, acetate and butyrate prevent oxidative and nitrosative stress (53) and recently, we found that butyrate protects pancreatic beta cells from IL-1β-induced impairment of insulin secretion and decreased insulin content (52). Transcriptomic analysis showed that the protective effects are particularly associated with downregulation of inflammatory genes, suggesting an anti-inflammatory role of butyrate in beta cells (52). However, the molecular mechanisms by which butyrate may mediate lower inflammation in beta cells are unknown. To elucidate the underlying mechanisms, in the present study, we investigated the effects of butyrate on inflammatory gene expression with focus on *Nos2* transcription and the NF-κB signaling pathway in mouse islets and the insulin-secreting cell line INS-1E.

## Results

### Butyrate inhibits IL-1β-induced expression of inflammatory genes in mouse islets and INS-1E cells

**Figure 1:**
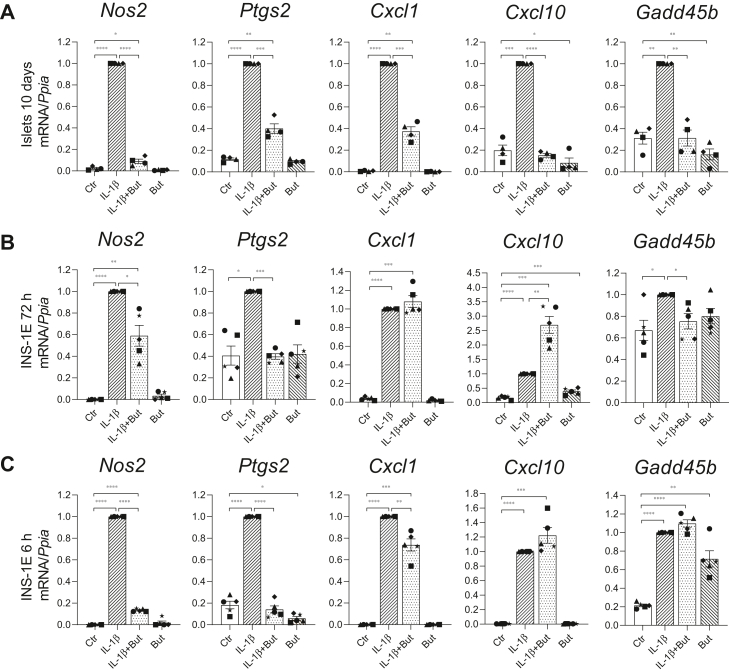
**Inflammatory gene expression in mouse islets and INS-1E cells.***A*, mouse islets were exposed to IL-1β (50 pg/ml) for 10 days and/or butyrate (But, 0.2 mM) or left unexposed (Ctr). INS-1E cells were cultured for 72 h (*B*) or 6 h (*C*) in presence or absence of IL-1β (12.5 pg/ml) and/or butyrate (0.4 mM) or left unexposed. Total RNA was extracted, and relative mRNA expression levels were analyzed using RT-qPCR. Expression levels were normalized to the expression of the housekeeping gene *Ppia* and are shown as fold increase relative to IL-1β-exposed islets/cells. Bars show means ± SEM of n = 4 to 5. ∗*p* < 0.05, ∗∗*p* < 0.01, ∗∗∗*p* < 0.001, ∗∗∗∗*p* < 0.0001. IL-1β, interleukin-1β.

To investigate the anti-inflammatory potential of butyrate in mouse islets and INS-1E cells, we measured the expression of selected inflammatory genes by RT-qPCR. As shown in Figure 1*A*, long-term exposure (10 days) of mouse islets to a noncytotoxic concentration of IL-1β (50 pg/ml) strongly increased the gene expression of the inflammation-associated enzymes *Nos2* (encoding iNOS) and *Ptgs2* (encoding cyclooxygenase 2), the chemokines *Cxcl1* and *Cxcl10* and the stress responder *Gadd45b*. In presence of butyrate (0.2 mM), the IL-1β-induced expression of *Cxcl10* and *Gadd45b* mRNAs in islets was completely prevented and more than 60% reduced for *Nos2*, *Ptgs2*, and *Cxcl1* mRNAs. Butyrate alone also significantly reduced the basal expression of *Cxcl10* and *Gadd45b* (Fig. 1*A*). At the protein level, IL-1β-induced CXCL1 and tumor necrosis factor-alpha secretion into the medium was also decreased in islets co-exposed to butyrate, whereas IL-6 protein levels were unaffected by butyrate in IL-1β-exposed islets (Fig. S1).

Mouse islets are clusters of different cell types, so in order to investigate the direct effect of butyrate on IL-1β-induced beta cell dysfunction, we used the insulin-secreting cell line INS-1E. INS-1E cells were exposed to IL-1β (12.5 pg/ml) for 3 days, which is known to impair glucose-stimulated insulin release (52). We found that the expression of inflammatory genes was significantly increased compared to unexposed cells (Fig. 1*B*). When cells were co-exposed to IL-1β and butyrate (0.4 mM), we observed that the expression of *Nos2*, *Ptgs2*, and *Gadd45b* was significantly reduced (Fig. 1*B*). Butyrate did not downregulate the IL-1β-induced expression of the chemokines *Cxcl1* and *Cxcl10* (Fig. 1*B*) in INS-1E cells in contrast to what was observed in islets. IL-1β-induced *Cxcl10* mRNA was even further upregulated by butyrate (Fig. 1*B*).

Since several feedback and regulatory events may influence IL-1β-regulated gene expression after 3 days, we also investigated the early effects after 6 h exposure to IL-1β and butyrate. After 6 h, butyrate reduced IL-1β-induced expression of *Nos2* and *Ptgs2* by 80% and *Cxcl1* by 25%, while the expression of *Cxcl10* and *Gadd45b* was not affected (Fig. 1*C*). Together, these results show that in mouse islets, butyrate partly or completely prevents the expression of inflammatory genes induced by IL-1β and that the anti-inflammatory effect of butyrate in INS-1E cells is gene-specific and time-dependent.

### Butyrate inhibits IL-1β-induced *Nos2* promoter activity, iNOS expression, and NO production

**Figure 2:**
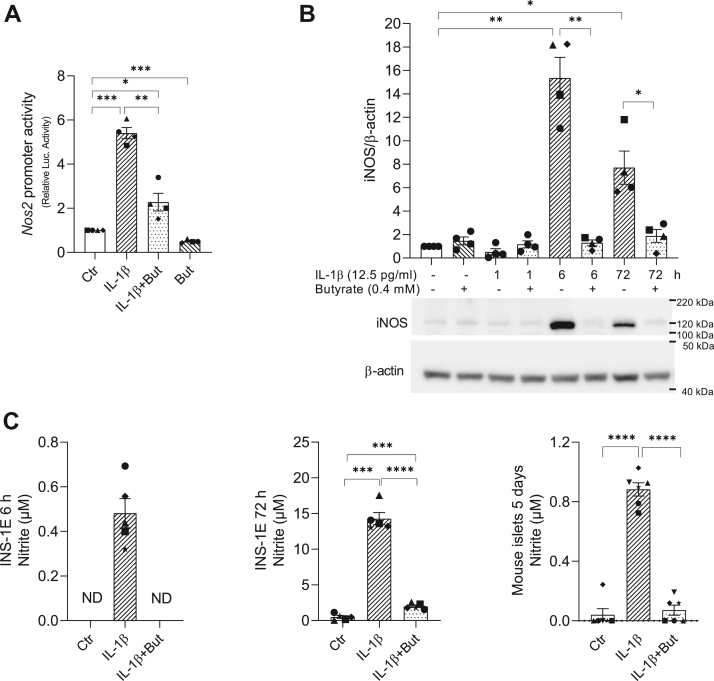
***Nos2* promoter activity, iNOS protein level, and NO production in INS-1E cells and mouse islets.***A*, INS-1E cells were transiently co-transfected with a *Nos2* promoter-luciferase plasmid and a Renilla plasmid to control for transfection efficiency for 4 h. The following day, cells were exposed to IL-1β (12.5 pg/ml) for 6 h and/or butyrate (But, 0.4 mM) or left unexposed (Ctr). Luciferase activity is shown after normalizing for Renilla luciferase activity. Data are shown as fold increase relative to Ctr. *B*, iNOS protein expression measured in whole cell extracts from INS-1E cells using Western blotting. Cells were exposed to IL-1β for 1, 6, or 72 h with or without butyrate. Cells exposed to IL-1β for 1 h were pre-exposed to butyrate for 1 h. Cells exposed to butyrate alone were exposed for 2 h. A representative blot is shown, and β-actin was used as loading control. Band intensities were quantified using Image Studio, and data are shown as fold increase relative to Ctr. *C*, NO production was measured as nitrite accumulation in culture medium from INS-1E cells or mouse islets (50 islets/ml) using Griess reagent. Cells were cultured for 6 or 72 h. Mouse islets were exposed to IL-1β (50 pg/ml) with or without butyrate (0.2 mM) for 5 days or left unexposed. Bars show means ± SEM of n = 4 to 6. ∗*p* < 0.05, ∗∗*p* < 0.01, ∗∗∗*p* < 0.001, ∗∗∗∗*p* < 0.0001. iNOS, inducible nitric oxide synthase; IL-1β, interleukin-1β; NO, nitric oxide; ND, not detectable.

To get insight into the mechanisms by which butyrate inhibits inflammatory gene expression, we focused on the effect of butyrate on *Nos2* transcription in INS-1E cells. *Nos2* encodes iNOS, an enzyme that produces NO, which has detrimental effects on beta cell function (15, 24, 25). To investigate whether butyrate inhibited IL-1β-induced *Nos2* mRNA expression at the level of transcription, INS-1E cells were transiently transfected with a plasmid containing the *Nos2* promoter fused to a luciferase reporter gene. Cells were then exposed to IL-1β, butyrate, or the combination for 6 h as other groups have shown a maximum induction of *Nos2* expression by IL-1β at this time point (55). We found that IL-1β increased *Nos2* promoter luciferase activity, but in presence of butyrate, the IL-1β-induced response was significantly reduced by 60% (Fig. 2*A*). Butyrate alone also decreased basal promoter activity (Fig. 2*A*). Moreover, IL-1β-induced iNOS protein expression was inhibited by butyrate after both 6 and 72 h (Fig. 2*B*). NO production, as measured by nitrite levels in the cell culture medium, was significantly induced by IL-1β after 6 and 72 h exposure and was significantly reduced by butyrate (Fig. 2*C*). In mouse islets, a similar effect of butyrate on IL-1β-induced NO formation was observed after 5 days exposure (Fig. 2*C*). Together, these results show that butyrate downregulates IL-1β-induced *Nos2* promoter activity, which leads to decreased iNOS protein level and NO production.

### Butyrate does not inhibit IL-1β-induced IκBα degradation and NF-κB p65 nuclear accumulation

**Figure 3:**
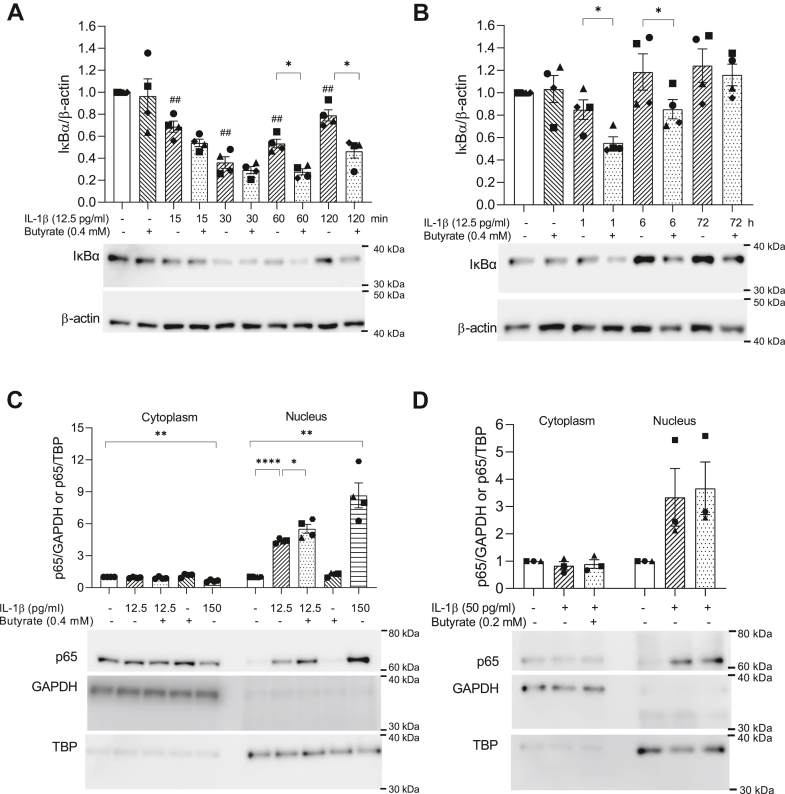
**IκBα protein levels and subcellular localization of NF-κB p65 in INS-1E cells and mouse islets.***A* and *B*, IκBα protein levels measured in whole cell extracts from INS-1E cells using Western blotting. Cells were exposed to IL-1β with or without butyrate (But) for various time points. Cells exposed to IL-1β for less than 6 h were preexposed to butyrate for 1 h. Cells exposed to butyrate alone were exposed for 1 h. Representative blots are shown, and β-actin was used as loading control. Band intensities were quantified using Image Studio and bars show means ± SEM of n = 4. Data are shown as fold increase relative to unexposed cells (Ctr). *C–D*, NF-κB p65 in cytoplasmic and nuclear extracts from INS-1E cells (*C*) and mouse islets (*D*) analyzed by Western blotting. INS-1 E cells or mouse islets were exposed to IL-1β for 1 h with or without 1 h pre-exposure to butyrate. A positive control exposed to 150 pg/ml IL-1β was included. GAPDH and TBP served as loading controls for cytoplasmic and nuclear fractions, respectively. Representative blots are shown. Band intensities were quantified using Image Studio. Data are shown as fold increase relative to Ctr and bars show means ± SEM of n = 3 to 4. ∗*p* < 0.05, ∗∗*p* < 0.01, ∗∗∗∗*p* < 0.0001. ##*p* < 0.01 *versus* Ctr. IL-1β, interleukin-1β; NF-κB, nuclear factor-κB.

The activation of NF-κB plays a key role in IL-1β-mediated signaling and inflammation (13). Therefore, we investigated whether butyrate regulates inflammatory gene expression *via* inhibition of NF-κB activation. As the activation of NF-κB requires IκBα degradation to allow translocation of NF-κB p65 to the nucleus (28), we first measured IκBα protein levels by Western blotting after 15 min to 72 h exposure to IL-1β with or without butyrate (Fig. 3, *A* and *B*). IκBα degradation was evident after 15 to 30 min exposure to IL-1β followed by reappearance after 60 min (Fig. 3*A*). IκBα levels returned to that of unexposed cells by 6 h (Fig. 3*B*), likely as a result of the well-established NF-κB-induced expression of IκBα. One hour pre-exposure to butyrate did not affect the IL-1β-induced degradation at early time points (15–30 min), neither did butyrate alone (Fig. 3*A*). In contrast, in cells exposed to IL-1β and butyrate, the reappearance of IκBα was first detected after 120 min and back to basal levels after 72 h, indicating that butyrate delays IκBα reappearance (Fig. 3, *A* and *B*).

To investigate mechanisms leading to the acute effects, we set to avoid feedback regulation by investigating the downstream components of the NF-κB signaling pathway in cells exposed to IL-1β for 1 h with or without 1 h pre-exposure to butyrate. Nuclear translocation of the NF-κB subunit p65 was induced by IL-1β in INS-1E cells and was slightly increased by butyrate (Fig. 3*C*). Butyrate alone did not promote p65 nuclear accumulation (Fig. 3*C*). A positive control exposed to 150 pg/ml IL-1β was included. Similar effects were observed in mouse islets exposed to IL-1β with or without butyrate (Fig. 3*D*). Together, these results show that butyrate does not inhibit NF-κB activity upstream of p65 nuclear translocation and the translocation itself.

### Butyrate affects IL-1β-induced NF-κB DNA binding in the context of native chromatin

**Figure 4:**
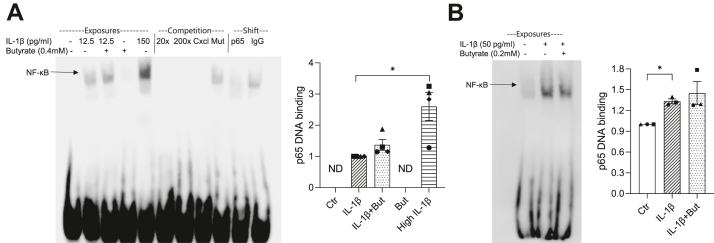
**NF-κB p65 DNA binding activity in INS-1E cells and mouse islets.** INS-1E cells (*A*) or mouse islets (*B*) were exposed to IL-1β for 1 h with or without 1 h preexposure to butyrate (But) or left unexposed (Ctr). A positive control exposed to 150 pg/ml IL-1β was included. NF-κB p65 DNA binding activity was measured using EMSA. Nuclear extracts were incubated with a probe containing a κB site of the *Nos2* promoter. Competition assays were performed with extracts from IL-1β-exposed INS-1E cells using 20 or 200 times excessive nonlabeled probe, a probe with a κB site within the *Cxcl1* promoter and a mutated probe (Mut). Supershifts were performed with anti-p65 and IgG antibodies. DNA–protein complexes were resolved on DNA retardation gels. Representative blots are shown. Band intensities were quantified using Image Studio. Data are shown as fold increase relative to IL-1β-exposed cells or Ctr and bars show means ± SEM of n = 3 to 4. ∗*p* < 0.05. ND, not detectable. EMSA, electrophoretic mobility shift assays; IL-1β, interleukin-1β; NF-κB, nuclear factor-κB.

Following nuclear translocation, NF-κB binds to specific genomic sequences in target gene promoters. To analyze NF-κB binding activity, electrophoretic mobility shift assays (EMSAs) were performed using nuclear extracts and probes containing a κB site from the *Nos2* promoter. IL-1β exposure promoted protein binding to the probe, as a specific DNA–protein complex appeared in extracts from INS-1E cells exposed to IL-1β (Fig. 4*A*). The band intensity was diminished by super-shift competition with an anti-p65 antibody, indicating the presence of NF-κB p65 in the complex. The binding specificity was confirmed as unlabeled probes and a probe containing a κB site of the *Cxcl1* promoter outcompeted the band, whereas no competition was observed with a probe containing a mutated κB site. In extracts from cells exposed to both IL-1β and butyrate, a DNA–protein complex similar to that obtained with extracts from cells exposed to IL-1β alone was observed, indicating that butyrate does not influence the binding to the κB site. Butyrate alone did not promote NF-κB binding (Fig. 4*A*). In mouse islets, IL-1β also increased NF-κB binding activity, which was unaffected by butyrate (Fig. 4*B*).

**Figure 5:**
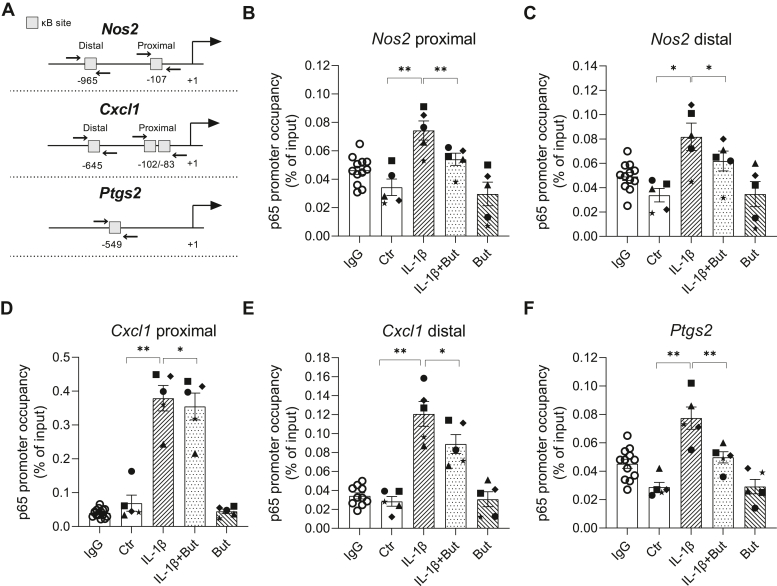
**Recruitment of NF-κB p65 to κB sites in the *Nos2*, *Cxcl1* and *Ptgs2* promoters in INS-1E cells.***A*, schematic showing the location of the targeted NF-κB binding sites in the promoters relative to the transcription start site (+1). Primers used for amplification of immunoprecipitated chromatin are indicated with *arrows*. *B–F*, INS-1E cells were exposed to IL-1β (12.5 pg/ml) for 1 h and/or butyrate (But, 0.4 mM) for 2 h or left unexposed (Ctr). ChIP assays were performed with an antibody that immunoprecipitated p65 or IgG as a negative control. Distal and proximal κB sites in the *Nos2* (*B* and *C*) or *Cxcl1* (*D–E*) and *Ptgs2* (*F*) promoters were targeted for amplification in the recovered DNA by qPCR. For the IgG control, data were pooled from three independent experiments. Data are shown as percentage of input DNA and bars show means ± SEM of n = 5. ∗*p* < 0.05, ∗∗*p* < 0.01. ChIP, chromatin immunoprecipitation; IL-1β, interleukin-1β; NF-κB, nuclear factor-κB.

Although IL-1β-induced p65 binding to synthetic DNA was unaffected by butyrate, it is possible that the binding differs in the context of native chromatin. To test this, we performed chromatin immunoprecipitation (ChIP) followed by qPCR for detection of p65 binding to proximal and distal κB sites within the *Nos2* promoter (See Fig. 5*A* for location of the κB sites). In INS-1E cells, IL-1β increased the recruitment of p65 to both the proximal and distal κB site within the *Nos2* promoter region, and butyrate reduced the binding by approximately 25% (Fig. 5, *B* and *C*). To confirm this observation for other IL-1β target genes, we investigated the recruitment of p65 to the *Cxcl1* and *Ptgs2* promoters known to contain κB sites (Fig. 5, *D*–*F*). Consistently, IL-1β increased the occupancy of p65 at these promoters, which was inhibited by butyrate (6% for *Cxcl1* proximal κB site (D), 26% for *Cxcl1* distal κB site (E) and 35% for *Ptgs2* (F)) but still significantly increased compared to unexposed cells. Together, these results show that butyrate does not affect the binding of p65 to synthetic DNA, but in the context of native chromatin, butyrate impairs p65 DNA binding.

### Butyrate reduces IL-1β-induced recruitment of RNA polymerase II to inflammatory gene promoters

**Figure 6:**
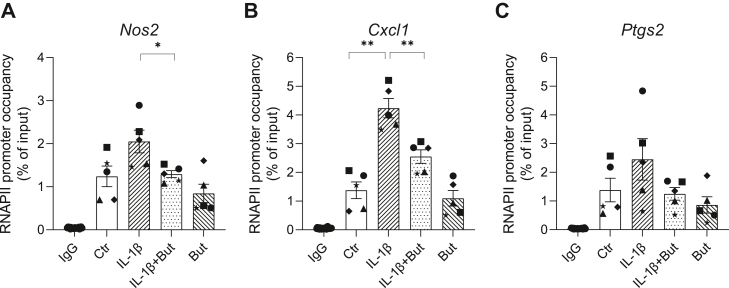
**Recruitment of RNA polymerase II to *Nos2*, *Cxcl1,* and *Ptgs2* promoters in INS-1E cells.** INS-1 E cells were exposed to IL-1β (12.5 pg/ml) for 1 h and/or butyrate (But, 0.4 mM) for 2 h or left unexposed (Ctr). ChIP assays were performed with an antibody that immunoprecipitated RNA polymerase II or IgG as a negative control. Regions of the *Nos2*, *Cxcl1*, and *Ptgs2* promoters near the transcription start sites were targeted for amplification in the recovered DNA by qPCR. For the IgG control, data were pooled from three independent experiments. Data are shown as percentage of input DNA and bars show means ± SEM of n = 5. ∗*p* < 0.05, ∗∗*p* < 0.01. ChIP, chromatin immunoprecipitation; IL-1β, interleukin-1β.

To investigate whether butyrate’s regulation of p65 DNA binding is accompanied by changes in the recruitment of RNA polymerase II (RNAPII) to target promoters, ChIP assays were performed with an anti-RNAPII antibody after IL-1β and butyrate exposure. We found that IL-1β increased the RNAPII occupancy near the transcription start sites at the *Cxcl1*, *Nos2*, and *Ptgs2* promoters (Fig. 6), consistent with the induction of these mRNAs (Fig. 1). Butyrate significantly reduced the IL-1β-induced recruitment of RNAPII to the *Nos2* (Fig. 6*A*) and *Cxcl1* (Fig. 6*B*) promoter by 37 and 40%, respectively. Although not statistically significant, a similar effect of butyrate was observed at the *Ptgs2* promoter (Fig. 6*C*). These results are consistent with the inhibitory effects of butyrate on IL-1β-induced expression of these genes (Fig. 1).

### Butyrate increases acetylation of NF-κB p65 and histone H4

**Figure 7:**
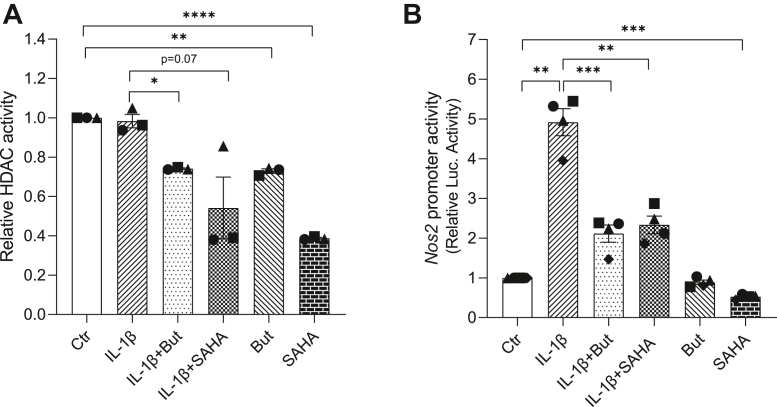
**HDAC activity and *Nos2* promoter activity in INS-1E cells.** HDAC activity (*A*) and *Nos2* promoter reporter-luciferase activity (*B*) in INS-1E cells exposed to IL-1β (12.5 pg/ml) for 1 h and/or butyrate (0.4 mM) or SAHA (0.5 μM) for 2 h. Ctr was left unexposed. Data are shown as fold increase relative to Ctr, and bars show means ± SEM of n = 3 to 4. ∗*p* < 0.05. ∗∗*p* < 0.01, ∗∗∗*p* < 0.001, ∗∗∗∗*p* < 0.0001. HDAC, histone deacetylase; IL-1β, interleukin-1β; sAHA, suberoylanilide hydroxamic acid.

**Figure 8:**
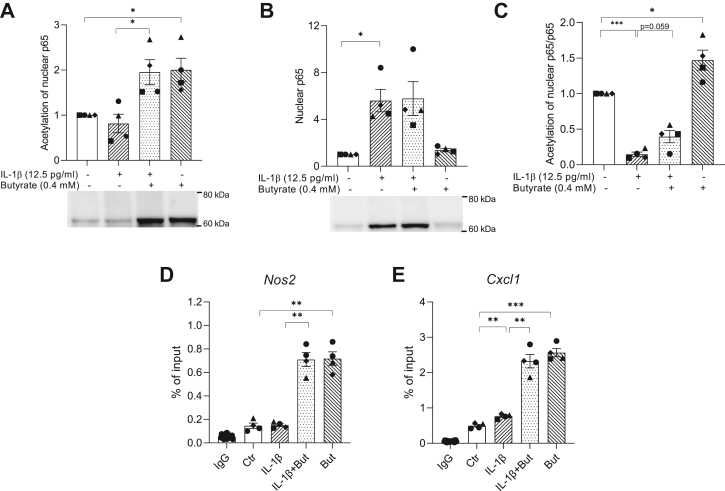
**Acetylation of NF-κB p65 and histone H4 in INS-1E cells.** INS-1 E cells were exposed to IL-1β (12.5 pg/ml) for 1 h and/or butyrate (0.4 mM) for 2 h or left unexposed (Ctr). *A*, acetylation of NF-κB p65. Nuclear extracts were immunoprecipitated using an anti-p65 antibody. Immunoprecipitated extracts were analyzed by Western blotting using an antibody directed against acetylated lysines (*A*) or p65 (*B*). The ratio of acetylated p65 over total nuclear p65 is shown in *C*. Representative blots are shown, and band intensities were quantified using Image Studio. Data are shown as fold increase relative to Ctr, and bars show means ± SEM of n = 4. ∗*p* < 0.05. *D–E*, acetylation of histone H4 at the *Nos2* (*D*) and *Cxcl1* (*E*) promoters. ChIP assays were performed with an antibody that immunoprecipitated acetylated (K5, K8, K12, K16) histone H4 or IgG as a negative control. For the IgG control, data were pooled from three independent experiments. Data are shown as percentage of input DNA and bars show means ± SEM of n = 4. ∗*p* < 0.05, ∗∗*p* < 0.01. NF-κB, nuclear factor-κB.

Butyrate is a known HDAC inhibitor (56) and since HDAC inhibition has been shown to regulate NF-κB activity (57, 58, 59), we hypothesized that the action of butyrate is mediated through inhibition of HDAC activity. To determine whether butyrate acts as a HDAC inhibitor in INS-1E cells, we measured HDAC activity *in situ* after 1 h exposure to IL-1β and/or 2 h exposure to butyrate or the pan-HDAC inhibitor suberoylanilide hydroxamic acid (SAHA). Butyrate and SAHA alone and in combination with IL-1β inhibited HDAC activity, whereas IL-1β alone did not affect HDAC activity (Fig. 7*A*). In order to examine the potential effect of HDAC inhibition on *Nos2* promoter activity, we analyzed the effect of SAHA using the *Nos2* reporter assay and found an inhibition similar to that observed by butyrate (Fig. 7*B*). To gain deeper insight into the mechanisms of action, we measured acetylation of lysine residues in p65 and histone H4. Acetylation of p65 was analyzed by Western blotting with a pan-acetyl-lysine antibody using p65 immunoprecipitated nuclear extracts. Butyrate significantly induced hyperacetylation of nuclear p65 both in presence and absence of IL-1β (Fig. 8*A*). The nuclear content of p65 in cells not exposed to IL-1β was low (Fig. 8*B*), indicating that the ratio of acetylated p65 over total nuclear p65 is lower in presence of IL-1β compared to in its absence (Fig. 8*C*). In cells co-exposed to butyrate and IL-1β, the fraction of acetylated p65 was higher compared to cells only exposed to IL-1β (Fig. 8*C*). Since recent studies have shown that hypoacetylation of histone H4 is associated with increased *Nos2* transcription (60, 61, 62), we investigated whether butyrate affected histone H4 acetylation near the *Nos2* and *Cxcl1* gene transcription start sites using ChIP assays with an anti-acetyl-histone H4 antibody. Butyrate markedly increased the acetylation of histone H4 both with and without IL-1β exposure (Fig. 8, *D* and *E*). The increase in acetylation was preserved after 3 days (Fig. S2). Together, these results show that butyrate enhances acetylation of both p65 and histone H4 within the *Nos2* and *Cxcl1* promoter region, suggesting a mechanism by which butyrate modulates the transcription of inflammatory genes.

## Discussion

Accumulating evidence indicates that the gut microbiota plays a role in metabolic health and T2D (32, 63). Specifically, butyrate, a short-chain fatty acid produced by certain gut bacteria, has anti-inflammatory properties (39, 40, 41, 42, 43, 44, 45) and affects glucose metabolism (32, 38, 64). Recently, we showed that butyrate protected beta cells from cytokine-induced dysfunction (52). In the current study, we further identify that butyrate inhibits IL-1β-induced inflammatory gene expression and NO production in mouse islets and INS-1E cells through suppression of NF-κB activation. In particular, we found that butyrate attenuated IL-1β-induced NF-κB binding and recruitment of RNAPII to inflammatory gene promoters in INS-1E cells, possibly mediated through changes in acetylation levels of NF-κB p65 and histone H4.

In our *in vitro* model, we exposed mouse islets and INS-1E cells to noncytotoxic low concentrations of IL-1β for several days to mimic the low-grade inflammatory environment surrounding beta cells in T2D. The low dose of IL-1β is sufficient to induce beta cell dedifferentiation, characterized by decreased expression of beta cell specific genes and reduced insulin secretion and content (12, 52). In this study, we showed that IL-1β-induced beta cell dysfunction coincides with increased expression of inflammatory genes, such as *Nos2*, *Cxcl1*, *Ptgs2*, and NO production. The precise function by which IL-1β alters beta cell function has not been established conclusively. However, it is likely that increased NO production by iNOS is at least partly responsible for the inhibitory effect, as other studies showed that inhibition of iNOS using small molecule inhibitors restores insulin secretion in rat (65) and human islets (23, 66). In addition, regulation of several cytokine-induced genes are secondary to NO formation (15), but NO independent mechanisms may also exist (26). Interestingly, butyrate markedly inhibited IL-1β-induced *Nos2* transcription, iNOS protein, and NO accumulation, suggesting a potential mechanism by which butyrate could prevent beta cell dysfunction. Similar inhibitory effects of butyrate on cytokine-induced iNOS expression and NO have also been reported in macrophages (39, 40, 44, 67), astrocytes (42) and chondrocytes (41, 68). Likewise, IL-1β-induced synthesis of prostaglandins produced by cyclooxygenase 2 (encoded by *Ptgs2*) is likely also to be inhibited in presence of butyrate, and *in vivo* reduced CXCL1 production may decrease recruitment and activation of immune cells and thereby attenuate local islet inflammation (18).

The anti-inflammatory properties of butyrate prompted us to examine the effect of butyrate on activation of the transcription factor NF-κB, which is required for *Nos2* expression (25, 69, 70) and involved in the regulation of several other inflammatory associated genes (71). Regulation of NF-κB activity occurs at multiple levels including, subcellular localization, posttranslational modifications of proteins as well as gene accessibility, and butyrate has been shown to modulate NF-κB activation at different levels in other cell types (39, 40, 41, 42, 43, 44, 45, 67, 72).

Subcellular localization of NF-κB is tightly regulated by inhibitory IκB proteins, *e.g.*, IκBα that in unstimulated cells sequester NF-κB in the cytoplasm. Upon IL-1β stimulation, IκBα is phosphorylated, ubiquitinated, and degraded by the proteasome, allowing NF-κB to translocate to the nucleus (28). Interestingly, butyrate had no significant effect on IL-1β-induced IκBα degradation at early time points, but the reappearance of IκBα was delayed. This delay has also been reported in cells exposed to HDAC inhibitors (48, 73), suggesting that butyrate potentially could act in a similar manner. Several mechanisms, not mutually exclusive, could explain the delayed reappearance of IκBα in presence of butyrate: (1) Decreased IκBα mRNA production as a result of reduced NF-κB transcriptional activity, (2) increased proteasome activity over time, or (3) prolonged activation of IκB kinase leading to increased phosphorylation of IκBα and thus persistent degradation.

In the nucleus, both posttranslational modifications of NF-κB and chromatin structural changes may determine gene activation by NF-κB, such as promoter accessibility, DNA binding, recruitment of the transcriptional machinery, and the duration of transcriptional activation. In butyrate-exposed cells, IL-1β-induced NF-κB binding and RNAPII recruitment to *Nos2*, *Cxcl1*, and *Ptgs2* promoters were significantly reduced in the context of native chromatin. Interestingly, EMSA showed that in a chromatin-free environment (*i.e.*, where nucleosomes are removed from the genomic region), IL-1β-induced NF-κB binding to κB sites was unaffected by butyrate, suggesting that impaired NF-κB DNA binding in INS-1E cells is due to chromatin remodeling promoted by butyrate rather than changes in posttranslational modifications of NF-κB p65. The fact that butyrate even further reduced the recruitment of RNAPII compared to the binding of p65 indicates that the transactivation potential of p65 is also impaired.

Butyrate is a potent HDAC inhibitor (56). Therefore, we speculated that its action could be mediated through inhibition of HDACs leading to increased acetylation of histones and/or nonhistone proteins such as p65. Indeed, we found that butyrate inhibited HDAC activity in INS-1E cells and that butyrate increased global acetylation of p65 and histone H4 near the transcription start sites of inflammatory genes. Exactly how, this hyperacetylation relates to inhibition of transcription needs further studies, but reversible acetylation and deacetylation events have been implicated in regulation of NF-κB transcriptional activity and regulation of *Nos2* gene expression (49, 57, 74). Furthermore, in this study, the HDAC inhibitor SAHA inhibited IL-1β-induced *Nos2* promoter activity similarly to what was observed using butyrate, supporting that acetylation events could likely play a role in the regulation of inflammatory gene expression by butyrate.

Acetylation of specific lysine residues within p65 by histone acetylases such as p300/CBP seems to have diverse consequences on NF-κB transcriptional activity. For example, acetylation of K122 and K123 reduces NF-κB DNA binding and deacetylation by HDAC3 is required for transcriptional activity (75, 76, 77). In contrast, acetylation of K310 is required for transcriptional activity, and acetylation of K221 enhances DNA binding, and deacetylation by HDAC3 results in increased association with IκBα and termination of activation (78, 79). Moreover, acetylation of p65 may regulate the recruitment of regulatory factors and RNAPII and possibly their activity could also be regulated by acetylation (80). p65 may also be phosphorylated which determines its association with histone acetylases or HDACs (81). Detection of specific acetylations is still challenging due to low abundance and lack of validated specific antibodies. Unfortunately, we were neither able to detect specific lysine residue acetylation of p65 nor the recruitment of HDAC3 and p300 to the gene promoters.

The importance of signal-induced changes in chromatin structure for NF-κB transcriptional activity has been demonstrated previously (27, 70, 71). Histone acetylation neutralizes positively charged lysines disabling the interaction with negatively charged DNA and thus loosening the chromatin structure to control the accessibility of DNA binding proteins (28). The large difference in histone H4 acetylation at the *Nos2* and *Cxcl1* promoter in INS-1E cells exposed to IL-1β alone *versus* IL-1β and butyrate suggests that the chromatin structure differs in these regions. Acetylation is rapidly induced after butyrate exposure. Wang *et al*. reported acetylation of histone H4 after 10 min in a sequential manner of specific residues (82), and we showed that the increased acetylation by butyrate also persisted after 3 days. One could speculate that these changes in histone acetylation by butyrate could affect gene accessibility and recruitment of p65 and RNAPII to the inflammatory gene promoters and thus reduce the IL-1β-induced transcriptional response. This is in line with studies showing that increased histone H4 acetylation is associated with suppression of *Nos2* transcription (60, 61, 62), although increased histone acetylation is generally associated with active transcription (83). However, some specific marks such as acetylation of H4K20 have been described to be enriched around the transcription start site of minimally expressed genes in humans (84). Acetylation of histone H3 is likely also involved and influenced by butyrate. Together, this suggests that the interpretation of increased global acetylation of p65 and histone H4 by butyrate is not straightforward and site specific and sequential acetylation may determine the effect on inflammatory gene expression. Whether acetylation proceeds transcription or is a consequence of transcription is also still debated (85).

Adding to the complexity, the expression of NF-κB target genes is differently regulated and is time, stimuli, and cell-type dependent. For example, we observed that the IL-1β-induced expression of *Cxcl1* and *Cxcl10* was significantly suppressed in presence of butyrate in mouse islets, but not in INS-1E cells. IL-1β-induced *Cxcl1* mRNA was only downregulated by butyrate after 6 h stimulation and not after 3 days. IL-1β-induced *Cxcl10* mRNA was even further upregulated by butyrate, suggesting that different mechanisms are involved in the regulation of inflammatory genes by butyrate. Differential effects of butyrate on inflammatory gene expression have been reported in IL-1β-stimulated intestinal epithelial cells (86) and chondrocytes (41). Blais *et al*. showed that genes unaffected by butyrate are more rapidly induced after IL-1β exposure and contains a higher number of κB sites compared to genes negatively regulated by butyrate (86). In contrast, Saccani *et al*. showed that gene-specific differences in LPS-induced NF-κB recruitment do not arise from variation in the number and affinity of κB sites but rather due to differences in the chromatin structure at promoter regions (71). Here, we detected that butyrate induced 3.5 times higher acetylation of histone H4 at the *Cxcl1* promoter compared to the *Nos2* promoter, highlighting that gene-specific changes exist. Yet another possibility is that the expression of some genes may require cooperative interactions with other transcription factors. Crosstalk between NF-κB pathway and mitogen-activated protein kinase pathway, which is also activated by IL-1β, has been reported (69, 87, 88). Furthermore, after exposure to butyrate and IL-1β for several days, the gene regulatory effects of butyrate are likely a mixture of direct and indirect effects. For example, IL-1β-induced *Gadd45b* expression is NO dependent (15), and therefore the stronger suppression of *Gadd45b* by butyrate after 3 days compared to 6 h may be a result of the inhibitory effects of butyrate on NO production.

In conclusion, our results show that butyrate plays an anti-inflammatory role in pancreatic beta cells. Butyrate downregulates inflammatory gene expression through inhibition of NF-κB signaling. Several mechanisms may account for the inhibitory effects of butyrate on IL-1β-induced NF-κB and RNAPII recruitment to inflammatory gene promoters, but it is likely that changes in the acetylation levels of NF-κB and histones play a role. Since inflammation contributes to beta cell dysfunction, blocking cytokine signaling by butyrate to prevent further activation of inflammatory mediators such as NO may be a successful way to preserve the function of insulin-producing beta cells.

## Experimental procedures

### Chemicals and reagents

A complete list with reagents, chemicals, software, instruments, and suppliers is found in Table S1.

### Cell culture and mouse islet isolation and culture

The insulin-secreting beta cell line INS-1E was maintained in RPMI 1640 medium with GlutaMAX supplemented with 10% heat inactivated fetal bovine serum, 100 U/ml penicillin and 100 μg/ml streptomycin (1% P/S), and 50 μM β-mercaptoethanol (complete medium). The cells were cultured at 37 °C in a humidified atmosphere with 5% CO_2_. Once a week, the cells were passaged. Cells for experiments were seeded in plates/dishes in duplicate for each condition at least 2 days prior to treatment, and media were changed before treatments. Cells were exposed to 12.5 pg/ml IL-1β, 0.4 mM butyrate, the combination, or left unexposed. At the end of the experiments, the cell confluence was 70 to 80%. For IL-1β exposures less than 6 h, butyrate was added 1 h prior to IL-1β.

Mouse islets from 10- to 12-week-old male C57BL/6NRj mice (Janvier) were isolated by perfusion *via* the common bile duct with Liberase solution (0.1 mg/ml Liberase TL, 0.1 mg/ml DNase I, 25 mM CaCl_2_ in HBSS with Ca^2+^ and Mg^2+^). The pancreases were excised, incubated in Liberase solution at 37 °C for 17 min, and the tubes were shaken vigorously. Digestion was stopped by ice-cold HBSS supplemented with 2.7 mM glucose and 0.3% bovine serum albumin, and the tubes were centrifuged at 200*g* for 3 min. Two washes were performed, and the islets were separated from exocrine tissue by filtration. The islets were handpicked and cultured in RPMI 1640 with GlutaMAX supplemented with 10% fetal bovine serum and 1 % P/S overnight. One day post isolation, the islets were transferred to RPMI 1640 with 2% human serum and exposed to 50 pg/ml IL-1β, 0.2 mM butyrate, the combination, or left unexposed for either 1 h or 10 days. For 10 days exposures, medium was changed on day 5. The animal experiments were approved by local veterinary ethics committee, and the mice were housed according to the Principles of Laboratory Care.

Cytokine and butyrate concentrations were chosen based on our previous studies (12, 52).

### Gene expression analysis by RT-qPCR

INS-1E cells (300,000 per well (for 3 days stimulation) or 600,000 per well (for 6 h stimulation)) were seeded in 6-well plates in 2 ml complete medium and incubated for 2 days. Following butyrate and IL-1β exposure, the cells were lysed with RA1 buffer (NucleoSpin kit), snap frozen, and stored at −80 °C. Total RNA was isolated using the NucleoSpin kit according to the manufacturer’s instruction.

Mouse islets were lysed in TRIzol, and RNA was extracted using Direct-zol RNA MiniPrep kit according to the manufacturer’s protocol.

cDNA was synthesized using the qScript cDNA Super mix kit. RT-qPCR reactions were performed using the TaqMan probes found in Table S2 and performed on the ABI PRISM 7900HT Sequence Detection System. Samples were determined in triplicate and expression normalized to the housekeeping gene *Ppia*. The relative expression levels were calculated from standard curves.

### NO and cytokine measurements

INS-1E cells were handled as described above, and medium was collected before lysis. Mouse islets (50 per condition in 1 ml medium) were exposed to IL-1β with or without butyrate for 10 days, and medium was collected when changed on day 5. NO was measured as accumulated nitrite since NO is rapidly oxidized into nitrite and nitrate. Briefly, 100 μl cell culture medium was mixed with 100 μl Griess reagent (equal amounts of 0.1% naphthylethylene diamine dihydrochloride in H_2_O and 1% sulphanilamide in 5% H_3_PO_4_) and incubated for 10 min. Absorbance was measured at 540 nm, and the nitrite concentration was calculated from a standard curve of sodium nitrite. IL-6, tumor necrosis factor-alpha, and CXCL1 were measured in medium from islets collected on day 5 using the Meso Scale Discovery (MSD) technology. The V-PLEX Pro-inflammatory Panel 1 (mouse) Multiplex Assay was used according to the manufacturer’s instructions. Plates were read on the MESO QuickPlex SQ 120 Imager.

### Gene reporter assay

INS-1E cells (80,000 per well) were seeded in 24-well plates with 1 ml complete medium in duplicate. After 3 days, medium was discarded and replaced by 400 μl serum- and antibiotic-free medium before the cells were transfected by lipofection using Lipofectamine 2000. Cells were cotransfected with 0.2 μg iNOS-luc-promoter plasmid and 0.2 μg of an internal control Renilla plasmid (pRL-TK) to control for transfection efficiency. The iNOS-luc-promoter plasmid contains the nucleotides -1002 to +132 of the *Nos2* promoter, which are required for maximal iNOS activation by IL-1β (16). The promoter was fused to a luciferase reporter gene as previously described (16). For each transfection, 1.5 μl Lipofectamine was mixed with plasmid DNA in Opti-MEM and incubated for 20 min at room temperature to allow DNA–Lipofectamine complexes to form. The complexes (100 μl) were added to the wells, and the cells were transfected for 4 h at 37 °C after which the medium was changed to 1 ml complete medium. The following day, the cells were stimulated with butyrate and IL-1β for 6 h. The cells were washed with PBS and incubated with lysis buffer (100 mM Tris-HCl, 50 mM Tris-base, 75 mM NaCl, 5 mM MgCl_2_, 0.25% Triton X-100) for 30 min shaking at room temperature. Firefly and Renilla luciferase activity were measured separately in 40 μl lysate using an in-house developed assay.

### Preparation of whole cell and nuclear/cytoplasmic extracts

Whole cell extracts were prepared for detection of IκBα and iNOS by Western blotting. Cells (300,000 or 450,000 per well) were seeded in 6-well plates in 2 ml complete medium and incubated for 2 or 3 days. IL-1β and butyrate were added as indicated for various time points ranging from 15 min to 3 days of stimulation. Cells were washed in HBSS and collected by centrifugation. The pellet was lysed in RIPA buffer (150 mM NaCl, 1% IGEPAL CA-630, 0.5% (w/v) sodiumdeoxycholate, 0.1% SDS, 50 mM Tris-HCl pH 8, 2 mM EDTA) supplemented with 10 mM β-glycerolphosphate and cOmplete Mini protease inhibitor cocktail (Roche). After 30 min incubation on ice, the lysates were centrifuged at maximum speed, and supernatants were collected. Protein content was determined by the BioRad DC Protein assay at 690 nm with a BSA standard.

Nuclear/cytoplasmic extracts were prepared for detection of translocation, DNA binding, and acetylation of NF-κB p65. Cells (2,800,000 per dish) or islets (∼400 per dish) were seeded in 100 mm dishes. On day 3, cells/islets were stimulated with IL-1β (1 h), butyrate (2 h), the combination, or left unexposed. Cells and islets were washed in HBSS and lysed in buffer A (20 mM Hepes pH 7.9, 1 mM EDTA, 1 mM MgCl_2_, 10 mM KCl, 20% glycerol, 1 mM DTT, 10 mM β-glycerolphosphate). Following 10 min incubation on ice, the lysate was collected and centrifuged at 2500 g for 5 min at 4 °C. The supernatant (cytosolic proteins) was saved at -80 °C. The pellet, containing nuclei, was resuspended in buffer B (buffer A supplemented with 400 mM NaCl) and incubated for 30 min on ice shaking. Nuclear proteins were collected by centrifugation at 15,000 g for 15 min at 4 °C, and supernatant was saved. Protein content was determined by the Bradford method at 620 nm with a BSA standard.

### Immunoprecipitation of NF-κB p65

INS-1E cell nuclear extracts, containing equal amounts of protein (∼200–300 μg) were diluted 1:4 in buffer A to decrease the salt concentration and were incubated overnight rotating with 1 μg p65 antibody. The immunocomplexes were collected by using prewashed Dynabeads M-280 Sheep Anti-Rabbit IgG. Following 2 h incubation, the beads were washed four times with PBS and subsequently the antibody complexes were eluted in NuPAGE LDS sample buffer with 0.1 M DTT and denatured at 80 °C for 10 min. The entire eluate was loaded on 10% Bis-Tris NuPAGE gels. Acetylation of lysine residues in p65 were detected by Western blotting as described in the following.

### Western blot analysis

Samples containing equal amounts of protein (5–20 μg) were mixed with NuPAGE LDS sample buffer with DTT (final concentration 0.1 M) and denatured at 80 °C for 10 min before loading on 10% Bis-Tris NuPAGE gels. After separation, the proteins were transferred to nitrocellulose membranes. The membranes were blocked 1 h with 5% (w/v) skim milk before the incubation with primary antibody overnight at 4 °C. A list of primary antibodies is found in Table S3. Horseradish peroxidase-linked anti-rabbit/mouse IgG were used as secondary antibodies and incubated 1 h at room temperature. Peroxidase activity was detected by chemiluminescence using the ECL Prime Western Blotting Detection Reagents and imaged on Odyssey Fc Imager. The intensity of each band was measured with the Image Studio Lite Ver 5.2.

### Electrophoretic mobility shift assay

Nuclear extracts from INS-1E cells and islets were prepared as described above. For the DNA binding assays, complimentary single-stranded unlabeled and 5′-biotinylated NF-κB p65 binding site DNA probes were annealed by heating at 90 °C for 10 min and slowly cooling down to room temperature. EMSA was performed using the LightShift Chemiluminescent EMSA kit. Briefly, reaction mixtures (20 μl) containing 1.5 to 10 μg nuclear extract protein were incubated with 20 fmol biotinylated probe in EMSA buffer (20 mM Hepes, 10 mM NaCl, 1 mM MgCl2, 1 mM EDTA, 10% glycerol, 1 mM DTT, 1 μg/μl Poly (dI/dC)) for 20 min at room temperature. Reaction products were separated on 6% DNA retardation gels, transferred to Biodyne B Nylon membranes, and fixed on the membranes by ultraviolet cross-linking. The biotin-labeled probes were detected with streptavidin-horseradish peroxidase and images captured by Odyssey Fc Imager. The intensity of each band was measured with the Image Studio Lite Ver 5.2. For competition analyses, 20/200-fold excess of unlabeled or 20 fmol mutant probe was included in the binding reactions. For p65 super-shift, 0.2 μg p65 antibody was preincubated with nuclear extract 30 min on ice before the binding reaction was performed as usual. The oligo sequences are found in Table S4.

### ChIP qPCR assays

INS-1E cells (3,200,000 per dish) were seeded in 150 mm culture dishes in 40 ml complete medium and cultured for 5 days. Cells were washed in HBSS and fixed in 1% formaldehyde in PBS for 10 min at room temperature. To stop crosslinking glycine was added to a final concentration of 125 mM for 5 min. After two washes in PBS, cells were harvested in SDS buffer (50 mM Tris-HCl (pH 8), 100 mM NaCl, 5 mM EDTA (pH 8), 0.2% NaN_3_, 0.5% SDS) supplemented with protease inhibitors. Nuclei were collected by centrifugation for 6 min at 1200*g*, and the pelleted nuclei were lysed in 1.3 ml ice cold IP buffer (100 mM Tris-HCl (pH 8), 100 mM NaCl, 5 mM EDTA (pH 8), 0.2% NaN_3_, 0.25% SDS, 2.5% Triton X-100 and protease inhibitors). Fragmentation was achieved by sonication (Bioruptor, Diagenode) to an average length of 200 to 500 bp (15 cycles of 30 s, high intensity). Chromatin was cleared by centrifugation at 20,000*g* for 15 min and diluted in IP buffer without SDS to a final conc of 0.1% SDS. For each ChIP, 15 μg of chromatin was used and incubated with 5 μg antibody at 4 °C overnight. Antibodies are found in Table S3. After immunoprecipitation, the immune complexes were recovered by adding 50 μl Dynabeads Protein G magnetic beads and incubated for 2 h at 4 °C. Beads were washed three times in low salt buffer (20 mM Tris-HCl (pH 8), 2 mM EDTA (pH 8), 1% Triton X-100, 0.1% SDS, 150 mM NaCl) and one wash with high-salt buffer (20 mM Tris-HCl (pH 8), 2 mM EDTA (pH 8), 1% Triton X-100, 0.1% SDS, 500 mM NaCl). Elution was performed in 120 μl 1% SDS and 0.1 M NaHCO_3_ followed by incubation at 65 °C for 6 h to reverse crosslinking. DNA was purified using Qiagen MinElute PCR purification kit and measured by real time PCR using SYBR Green. Primers are found in Table S5.

### HDAC activity assay

INS-1E cells (20,000 per well) were seeded in a 96-well plate. On day 3, cells were exposed to IL-1β for 1 h and/or butyrate or SAHA (0.5 μM) for 2 h, and HDAC activity was measured using the *In situ* HDAC Activity Fluorometric Assay kit according to the manufacturer’ instructions. The cells were exposed to media containing the HDAC substrate for 2 h, and treatments were continued throughout the period. The lysates were transferred to a 96-black well plate with a clear bottom and the fluorescence excitation/emission was read at 368/442 nm. HDAC enzyme activity was expressed relative to the unexposed cells.

### Statistical analysis

GraphPad Prism was used to perform statistical analysis to compare two independent groups using a two-tailed paired *t* test. All data are expressed as mean ± standard error of mean (SEM) of n independent experiments.

## Data availability

All data are contained within the manuscript and Supporting information.

## Supporting information

This article contains supporting information.

## Conflict of interests

The authors declare that they have no conflicts of interest with the contents of this article.
