# Supplementary material for: Butyrate inhibits IL-1β-induced inflammatory gene expression by suppression of NF-κB activity in pancreatic beta cells
Source: J Biol Chem. 2022 Jul 31;298(9):102312. doi: 10.1016/j.jbc.2022.102312 (PMC9428856; doi:10.1016/j.jbc.2022.102312)
Supplement: Supporting information [file mmc1.pdf]

Butyrate inhibits IL-1β-induced inflammatory gene expression by suppression of NF-κB activity in pancreatic beta cells

Signe Schultz Pedersen, Michala Prause, Kristine Williams, Romain Barrès, Nils Billestrup

Supporting Information

List of material included:

<u>Figures:</u>

Figure S1. Cytokine levels in medium from mouse islets.
Figure S2. Acetylation of histone H4 at the transcription start sites of the *Nos2* and *Cxcl1* promoter.

<u>Tables:</u>

Table S1. Reagents, chemicals, software and instruments used in the study
Table S2. TaqMan probes used for gene expression analysis
Table S3. Antibodies used for western and immunoprecipitation
Table S4. Probes used for EMSA
Table S5. Primers used for ChIP-qPCR analysis

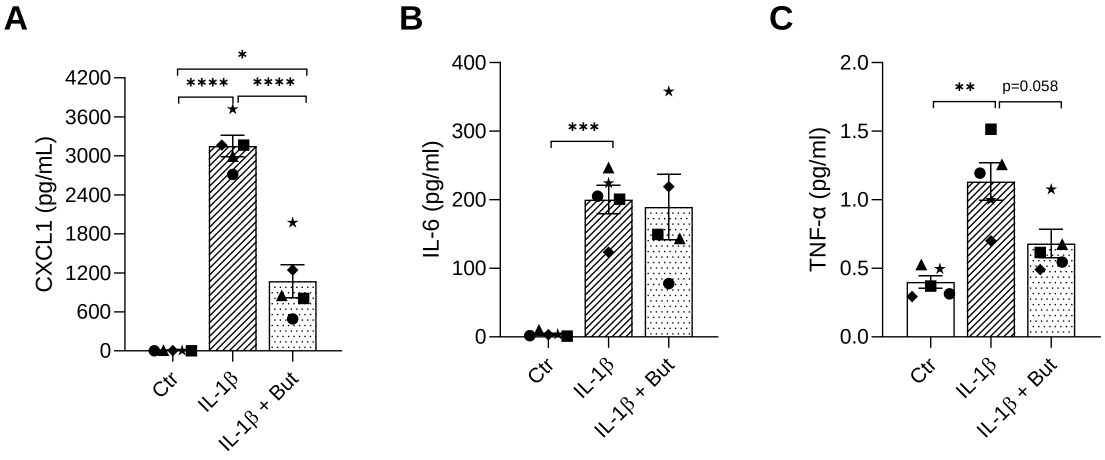

Figure S1. Cytokine levels in medium from mouse islets.

Mouse islets were exposed to IL-1β (50 pg/ml) and/or butyrate (But, 0.2mM) for 5 days or left unexposed (Ctr). Cytokines were measured in the culture medium (50 islets/mL medium). Bars show means ± SEM of n=5. *p<0.05, **p<0.01, ***p<0.001, ****p<0.0001.

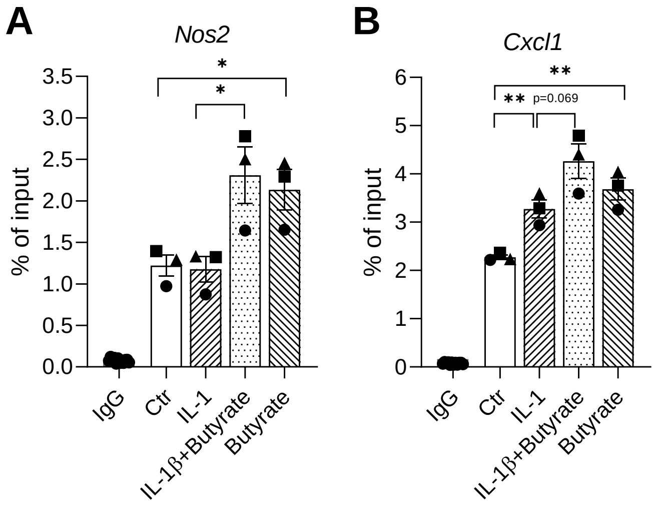

Figure S2. Acetylation of histone H4 at the transcription start sites of the *Nos2* and *Cxcl1* promoter. INS-1E cells were exposed to IL-1β (12.5 pg/ml) and/or butyrate (But, 0.4mM) for 72 h or left unexposed (Ctr). ChIP assays were performed using antibodies against acetylated (K5, K8, K12, K16) histone H4 or IgG as a negative control. Regions at the transcription start site of the *Nos2* and *Cxcl1* promoters were amplified in the recovered DNA by qPCR. Data are shown as percentage of input DNA and bars show means ± SEM of n=3. *p<0.05, **p<0.01.

Table S1. **Reagents, chemicals, software and instruments used in the study**

Reagent/resource	Supplier	Product #
**Media**		
RPMI 1640 medium with GlutaMAX	Gibco, Thermo Fisher Scientific	61870-010
Penicillin/streptomycin	Gibco, Thermo Fisher Scientific	15140-122
β-mercaptoethanol	Gibco, Thermo Fisher Scientific	31350-010
Fetal bovine serum	Gibco, Thermo Fisher Scientific	10276-106
Human serum	Biowest	S4190-100
**Experimental models**		
INS-1E	Gift from Claes Wollheim, (University of Geneva, Switzerland)	
C57BL/6NRj	Janvier Labs, Le Genest-Saint-Isle, France	
**Mouse islet isolation**		
Liberase TL	Roche	5401020001
**Treatments**		
Recombinant Mouse IL-1β	BD Biosciences	554577
Sodium butyrate	Sigma-Aldrich	B5887
SAHA	Sigma-Aldrich	SML0061
**Gene expression**		
NucleoSpin kit	Macherey-Nagel	740955.5
TRI reagent	Sigma-Aldrich	T9424
Direct-zol RNA MiniPrep kit	Zymo Research	R2052
qScript cDNA Super mix kit	Quantabio	95048-025
TaqMan	Applied Biosystems	4444557
**Nitric oxide and cytokine measurements**		
naphthylethylene diamine dihydrochloride	Sigma-Aldrich	N-9125
sulphanilamide	Sigma-Aldrich	S-9251
V-PLEX Proinflammatory Panel 1 Mouse Kit	Meso Scale Discovery	K15048D
**Gene reporter assay**		
Lipofectamine 2000	Invitrogen	11668-027
iNOS-luc-promoter plasmid	Gift from Decio Eizirik (Universitet Libre de Bruxelles, Brussels, Belgium)	
Renilla plasmid pRL-TK	Promega	E2241
Opti-MEM	Gibco, Thermo Fisher Scientific	31985-062
**Western blotting and immunoprecipitation**		
cOmplete Mini protease inhibitor cocktail	Roche	11836153001
BioRad DC Protein assay	Bio-Rad	500-0114
Protein assay (Bradford method)	Bio-Rad	5000002

Dynabeads™ M-280 Sheep Anti-Mouse IgG	Invitrogen	11201D
Dynabeads™ M-280 Sheep Anti-Rabbit IgG	Invitrogen	11203D
LDS sample buffer	Invitrogen	NP0007
NuPAGE MOPS buffer	Novex	NP0001
10 % Bis-Tris NuPAGE gels	Invitrogen	NP0301BOX/ NP0316BOX
nitrocellulose membranes	Invitrogen	LC2001
Polyclonal rabbit anti-mouse Immunoglobulins HRP	Dako	P0260
ECL Rabbit IgG, HRP-linked whole Ab (from donkey)	Amersham	NA934V
ECL Prime Western Blotting Detection Reagent	Amersham	RPN2232
**EMSA**		
LightShift Chemilumininescent EMSA kit	Thermo Fisher Scientific	20148
DNA retardation gels (6 %)	Invitrogen	EC63652BOX
Biodyne™ B Nylon membranes	Thermo Fisher Scientific	77016
**ChIP-qPCR**		
Dynabeads Protein G	Invitrogen	10003D
Qiagen MinElute PCR purification kit	Qiagen	28004
Fast SYBR™ Green Master Mix	Applied Biosystems	4385612
**HDAC activity assay**		
In Situ HDAC Activity Fluorometric Assay Kit	Sigma-Aldrich	EPI003
**Software**		
Image studio lite version 5.2	LI-COR Biosciences	
GraphPad Prism version 9.3.1	www.graphpad.com	
**Instruments**		
ODYSSEY Fc Imager	LI-COR Biosciences	
ABI PRISM 7900HT Sequence Detection System	Applied Biosystems	
Bioruptor	Diagenode	
MESO QuickPlex SQ 120	Meso Scale Discovery	

Table S2. TaqMan probes used for gene expression analysis

Gene	Probe	
	**Mouse (islets)**	**Rat (INS-1E cells)**
Cxcl1	Mm04207460_m1	Rn00578225-m1
Cxcl10	Mm00445235_m1	Rn01413889_g1
Gadd45b	Mm00435123-m1	Rn1452530-g1
Nos2	Mm00440502-m1	Rn00561646_m1
Ptgs2	Mm00478374_m1	Rn01483828_m1
Ppia	Rn00690933_m1	Rn00690933_m1

TaqMan probes were purchased from Thermo Fisher Scientific, Copenhagen, Denmark.

Table S3. Antibodies used for western and immunoprecipitation

Antibody	Manufacturer	Catalogue number	Dilution
**Western blotting primary antibodies**			
IκBα	Santa Cruz	Sc-371	1:1000
iNOS	Biosciences Pharmingen	#610332	1:1000
NF-κB p65	Santa Cruz	Sc-372	1:5000
Acetyl-Lysine, clone 4G12	MilliporeSigma	#05-515	1:1000
β-actin	Abcam	Ab6276	1:50,000
GAPDH (D4C6R)	Cell Signaling	#97166	1:1000 or 1:5000
TBP	Cell Signaling	#8515	1:1000
**Western blotting secondary antibodies**			
Polyclonal rabbit anti-mouse Immunoglobulins HRP	Dako	P0260	
ECL Rabbit IgG, HRP-linked whole Ab (from donkey)	Amersham	NA934V	
**Chromatin immunoprecipitation**			
NF-κB p65 (D14E12)	Cell Signaling	#8242	1:100
Rpb1 CTD (4H8)	Cell Signaling	#2629	1:100
Acetyl-Histone H4	MilliporeSigma	#06-866	1:125
Normal Rabbit IgG	Cell Signaling	#2729	1:100

Table S4. Probes used for EMSA

	Probes
Nos2 NF-κB distal	5’ TATGCCAGGGGGATTTTCCCTCTCTCT 3’<br>5’ AGAGAGAGGGAAAATCCCCCTGGCATA 3’
Mutated *Nos2* NF-κB distal	5’ TATGCCAGGAGCCTTTTGCCTCTCTCT 3’<br>5’ AGAGAGAGGCAAAAGGCTCCTGGCATA 3’
Cxcl1 NF-κB prox	5’ CGGTTGTGGGGAAACACCCTGTGCTCC 3’<br>5’ GGAGCACAGGGTGTTTCCCCACAACCG 3’

NF-κB consensus binding sequence is underlined and mutated bases are marked in red. The probes were purchased from TAG Copenhagen, Copenhagen, Denmark.

Table S5. Primers used for ChIP-qPCR analysis

Gene	Primer	
	**Forward**	**Reverse**
Cxcl1 NF-κB prox	5’ GCGATGTCCTTTCCGGTTG 3’	5’ TCCGGCGAGCCCTTTTATG 3’
Cxcl1 NF-κB dist	5’ ATACCGGGAGTTTGGGAGTTC 3’	5’ CCAAGGTTAAGCCCCTGTTTAC 3’
Nos2 NF-κB prox	5’ GTCCATCGCGAATGAGCTA 3’	5’ TATACCCATCCACGCTCTGC 3’
Nos2 NF-κB dist	5’ TGGGATGATGAGTGGACCC 3’	5’ AGGGGAAAAGGAACAAACAGAG 3’
Ptgs2 TSS	5’ GAGGCGGAAAGACACAGTCA 3’	5’ AGTTTGACAACTGGCCGCTA 3’
Ptgs2 NF-κB prox	5’ AACTGTGTGCGTGCTCAGAG 3’	5’ CGGGATCCGAGATCCTAACT 3’

Primers were purchased from TAG Copenhagen, Copenhagen, Denmark.
